# Transmission of cytomegalovirus via breast milk in low birth weight and premature infants: a systematic review and meta-analysis

**DOI:** 10.1186/s12887-021-02984-7

**Published:** 2021-11-22

**Authors:** Xiaolin Hu, Wei Hu, Xuan Sun, Ling Chen, Xiaoping Luo

**Affiliations:** Pediatric Department, Tongji Hospital, Tongji Medical Collage, Huazhong University of Science and Technology, Wuhan, China

**Keywords:** Breast milk, Cytomegalovirus, Preterm infant, Infection transmission, meta-analysis

## Abstract

**Background:**

This study aimed to investigate the transmission of cytomegalovirus (CMV) via breast milk in low birth weight (LBW) and premature infants and its effects.

**Methods:**

PubMed, Medline, Cochrane Library, and Embase were searched for studies (without language and time restriction) published before March 27, 2020, that examined the effect of CMV transmitted by breast milk on LBW and premature infants. The rates of breast milk-acquired CMV infection, CMV-related symptoms, and CMV-related sepsis-like syndrome (CMV-SLS) in LBW and premature infants were pooled from each study.

**Results:**

Eighteen studies with 1920 LBW and premature infants were included. The pooled CMV infection rate from breast milk for infants fed untreated breast milk was significantly higher than those fed frozen breast milk [19.3, 95% confidence interval (CI) = 11.8–29.9% vs. 13.5, 95% CI = 8.0–22.0%, *P* < 0.01). Similarly, the pooled CMV infection rate for infants fed untreated breast milk was significantly higher than those with mixed feeding (*P* < 0.0001). The mixed feeding group had a significantly lower rate of CMV-related symptoms than the other groups (2.4%, *P* < 0.01).

**Conclusions:**

These findings suggested a higher CMV infection rate in LBW or premature infants fed untreated breast milk than other feeding groups. Studies on the long-term outcomes of CMV infection transmitted from breast milk are needed to address the optimal feeding practice.

**Supplementary Information:**

The online version contains supplementary material available at 10.1186/s12887-021-02984-7.

## Background

Cytomegalovirus (CMV) is a member of the beta-herpes virus subfamily and causes serious clinical consequences in premature infants [1, 2], including respiratory failure, neutropenia, thrombocytopenia, hepatomegaly, and septic syndromes [3, 4]. Over the past few years, breast milk from seropositive mothers has been considered the main source of postnatally acquired CMV infection in preterm infants [5, 6]. CMV infection is often diagnosed by polymerase chain reaction (PCR), cell culture, and identification of CMV-specific antibodies or intracellular viral proteins [2, 6].

Almost 96% of the seropositive mothers have CMV reactivation, defined as shedding of viable virus or the presence of CMV deoxyribonucleic acid (DNA) in breast milk [7]. Previous studies revealed that the transmission rate of CMV from CMV-positive breast milk to preterm infants ranges from 37 to 87% [5, 7, 8]. Moreover, preterm infants were at higher risk of CMV infection than full-term newborns [6]. In addition, poor outcomes such as abnormal laboratory findings to a sepsis-like syndrome and even neurologic sequelae were observed in preterm infants [9, 10].

A previous meta-analysis [11] reported that CMV-related sepsis-like syndrome (CMV-SLS) is relatively rare in infants with breast milk-acquired CMV infection. Still, recently, several studies [12, 13] on breast milk-acquired CMV infection in low birth weight (LBW) or preterm infants provided new evidence on this topic. In addition, some studies [13, 14] reported a strong association between breast milk-acquired CMV and CMV-SLS in LBW and premature infants. Thus, the most recent published studies make it possible to complete a new systematic review and meta-analysis with more statistical power. Therefore, a meta-analysis was conducted to investigate the effects of transmission of CMV via breast milk in LBW and premature infants.

## Methods

### Literature search

PubMed, Medline, Cochrane Library, and Embase were searched for relevant studies on the research topic published before March 27, 2020. The individual and combined key words such as “breastfeeding”, “breast milk”, “premature”, “preterm”, “low birth weight infants”, “cytomegalovirus”, and “CMV” were used for searching the literature. The detailed search strategy can be found in Supplementary Table 1. The search terms were kept as broad as possible to identify relevant publications. In addition, in order to include more potential studies, bibliographies of all relevant studies and reviews identified were searched, and also Google Scholar was searched for relevant studies. This current study was conducted according to the Preferred Reporting Items for Systematic Reviews and Meta-analysis guidelines (PRISMA) statement [15].

### Eligibility criteria

Studies that investigated the effects of transmission of CMV infection via breast milk in LBW and premature infants were included. The inclusion criteria were 1) observational studies reporting the results of transmission of CMV infection via breast milk, 2) study subjects were LBW and premature infants, 3) data could be obtained from the original studies, 4) studies published in English, and 5) studies with more detailed information were selected if the population was reported in duplicate.

Reviews, case reports, comments, animal experiments, studies in languages other than English, and unavailable studies were excluded from the analysis.

### Data extraction and definition

All relevant articles from the databases mentioned above were screened independently by two authors (XH and WH) to decide whether the studies could be included in the full-text analysis. The data and necessary information of the relevant studies were extracted using a standardized form independently by two reviewers (XH and WH), and a consensus was reached on all items by a discussion with a third reviewer (XL). Information such as study characteristics (e.g., author and year of publication, country, sample size, and study design), patient characteristics (e.g., weight and gestational age at birth, sex of infants, and nationality), CMV infection characteristics (e.g., methods for diagnosing congenital CMV infection and postnatal CMV infection among infants), breast milk characteristics (e.g., pasteurization, freezing, and no treatment), and infant characteristics (e.g., numbers of infants who acquired postnatal CMV and outcomes, birth weight, and corrected gestational age at the onset of CMV infection) were extracted from each study.

Congenital CMV infection was defined as viral culture or CMV nucleic acid testing positive in blood or urine samples obtained within 2 weeks after birth. Postnatal CMV infection was defined as a positive congenital CMV positive and with a previously documented negative result. Infants born before 37 weeks gestation are considered premature.

### Quality scoring of studies

The two reviewers mentioned above (XH and WH) assessed the methodological strength of all included studies independently using the Newcastle-Ottawa Scale (NOS) [16], a procedure for independently assessing the methodological quality of observational studies. The NOS includes three categories: 1) patient selection (three items), 2) comparability of two study arms (two items), and 3) assessment of the outcomes (two items).

Studies were given a maximum of one point for each item within the selection and exposure categories and a maximum of two points for comparability. Studies were graded based on an ordinal scoring scale, and the score ranged from 2 to 9 stars; 2 to 5 stars was considered poor quality and 6–9 stars high quality. In order to provide more information on the quality assessment, we also used the Critical Appraisal Skills Programme (CASP) cohort checklist for the included studies (available at: https://casp-uk.net).

### Statistical analysis

The estimated rates of breast milk-acquired CMV infection, CMV-related symptoms, and CMV-SLS in LBW were extracted from each included study. The estimated rates of breast milk-acquired CMV infection, CMV-related symptoms, and CMV-SLS in LBW and premature infants were pooled by rates and relevant 95% confidence intervals (CIs). Inverse variance methods with random-effects were conducted to pool the results of included studies. The standard heterogeneity test, *I*^*2*^ statistic, was used to assess the consistency of the effect sizes. It indicates the percentage of variability in effect estimates because of true between-study variance rather than within-study variance. Heterogeneity was categorized into with and without significant heterogeneity according to the values of *I*^*2*^ by ≥50 and < 50% [17], respectively. In order to explore the sources of heterogeneity, all enrolled studies were sequentially excluded to demonstrate the overall impact of individual studies, where *I*^*2*^ > 50% indicated significant heterogeneity. We also conducted random-effects meta-regression analyses to assess the impact of covariates on infection effects. Publication bias was assessed by Begg’s rank correlation [18] and Egger’s weighted regression methods [19]. Review Manager (version 5.3, The Cochrane Collaboration, Oxford, UK) was used for statistical analyses. The Begg’s and Egger’s tests were assessed by STATA 15.0 (Stata Corporation, College Station, TX, USA). A *P* value of < 0.05 was statistically considered significant for all analyses.

## Results

### Study selection

**Fig. 1:**
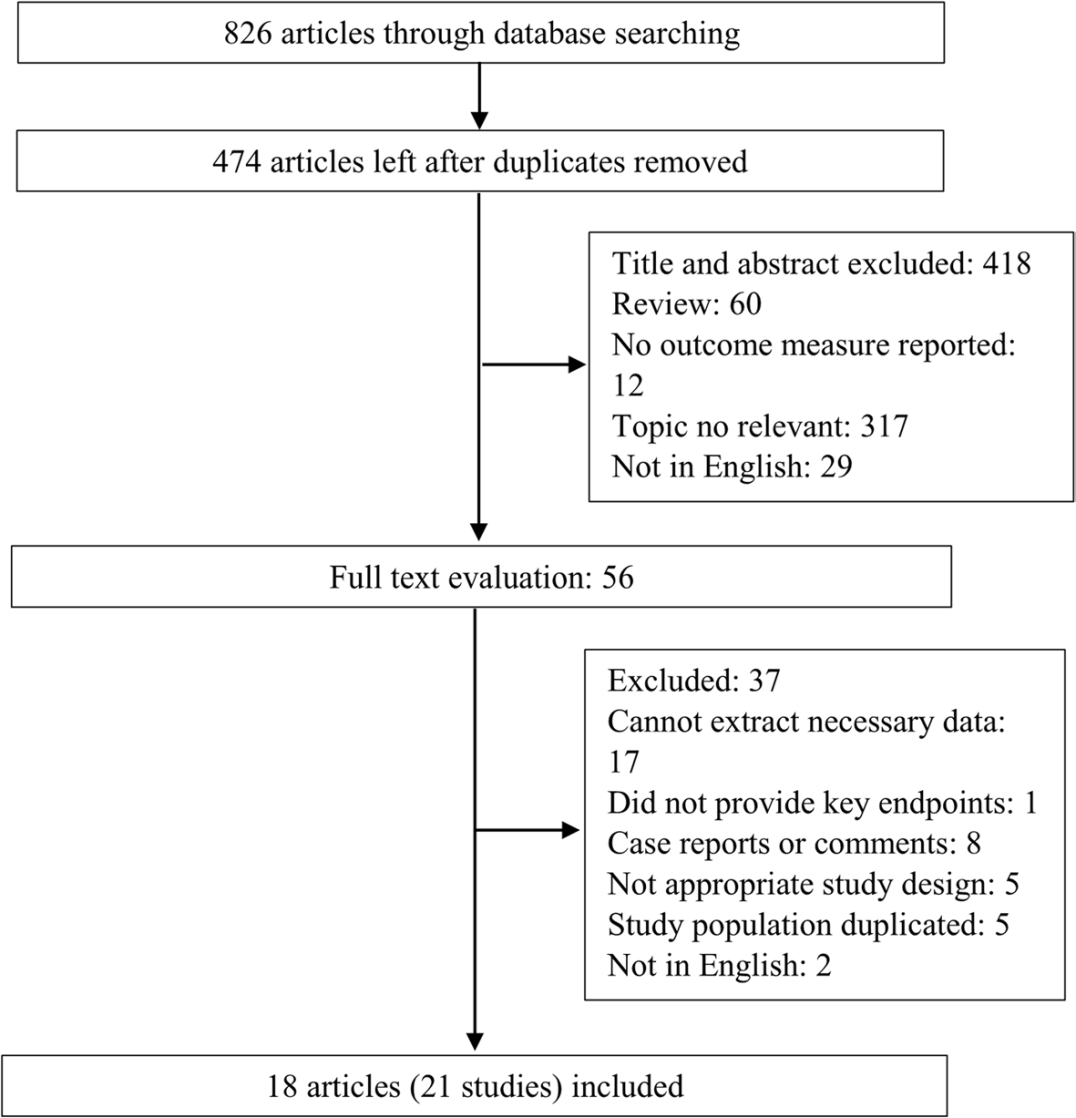
Flow chart of the study selection process

The study selection process is presented in Fig. 1. The systematic literature search yielded a total of 826 studies. Of these, 352 were excluded due to duplications, and the abstracts of 418 were initially reviewed. After careful reviewing, the full-length manuscripts of 56 studies were obtained. Following the inclusion and exclusion criteria, 18 studies [7, 12–14, 20–33] were finally included for data extraction and meta-analysis after excluding one study that used the same population [34].

### Study characteristics

The 18 studies included in this review included a total of 1920 LBW and premature infants. Two studies were divided into two studies according to the treatment method of the milk. The included studies were published between 2001 and 2019. The number of infants in each study ranged from 7 to 539. Most studies, except one, were prospective cohort studies [33]. Four of the included studies were conducted in Germany [12, 22, 26, 30], two in the United States of America [14, 31], two each in Brazil [13, 21], Japan [20, 33], Sweden [26, 32], Taiwan [21, 29], and Italy [28], and one each in Canada [23], Israel [25], and Korea [33]. Of the included studies, 17 used PCR, four used rt-PCR (real-time polymerase chain reaction), and one used immunofluorescence for CMV detection in infants. The inclusion criteria for infants were based on gestational age (GA, weeks) and/or body weight (BW, grams) of the infants. Most studies set 1500 g as the cut-off value for BW (ranged from 1000 to 1710 g of the included studies) and 32 weeks as the criteria for GA (ranged from 28 to 35 weeks). Most studies defined 21 days as the cutoff for testing for postnatally acquired CMV. The infants in 12 included studies were fed untreated breast milk, and in six studies were fed breast milk that was frozen at − 18 °C to − 20 °C for > 24 or 72 h. In seven studies, the infants were fed untreated breast milk in combination with frozen breast milk and/or pasteurized breast milk from donors. Seventeen studies reported the means or median of BW of infants, which ranged from 748 to 1300 g. The characteristics of the included studies and participants are summarized in Supplementary Tables 1 and 2. As shown in Supplementary Fig. 1, a total of 1598 mothers in the included studies were reported to be serologically CMV positive (IgG or IgM, or the combination of IgG and IgM). The pooled CMV positive rate of the included mothers was 77.6% (95%CI = 66.0–86.0%, *I*^*2*^ = 24%).

### Quality assessment of the studies

All eligible studies were of high quality (≥6 stars). Some of the included studies did not conduct adequacy of follow-up of their cohorts. Meanwhile, most studies enrolled outpatients, and the representativeness, therefore, was insufficient. The NOS scores for the eligible studies are presented in Supplementary Table 3. Similarly, CASP also indicated the high quality of all the included studies.

### CMV transmission to the preterm infant

**Fig. 2:**
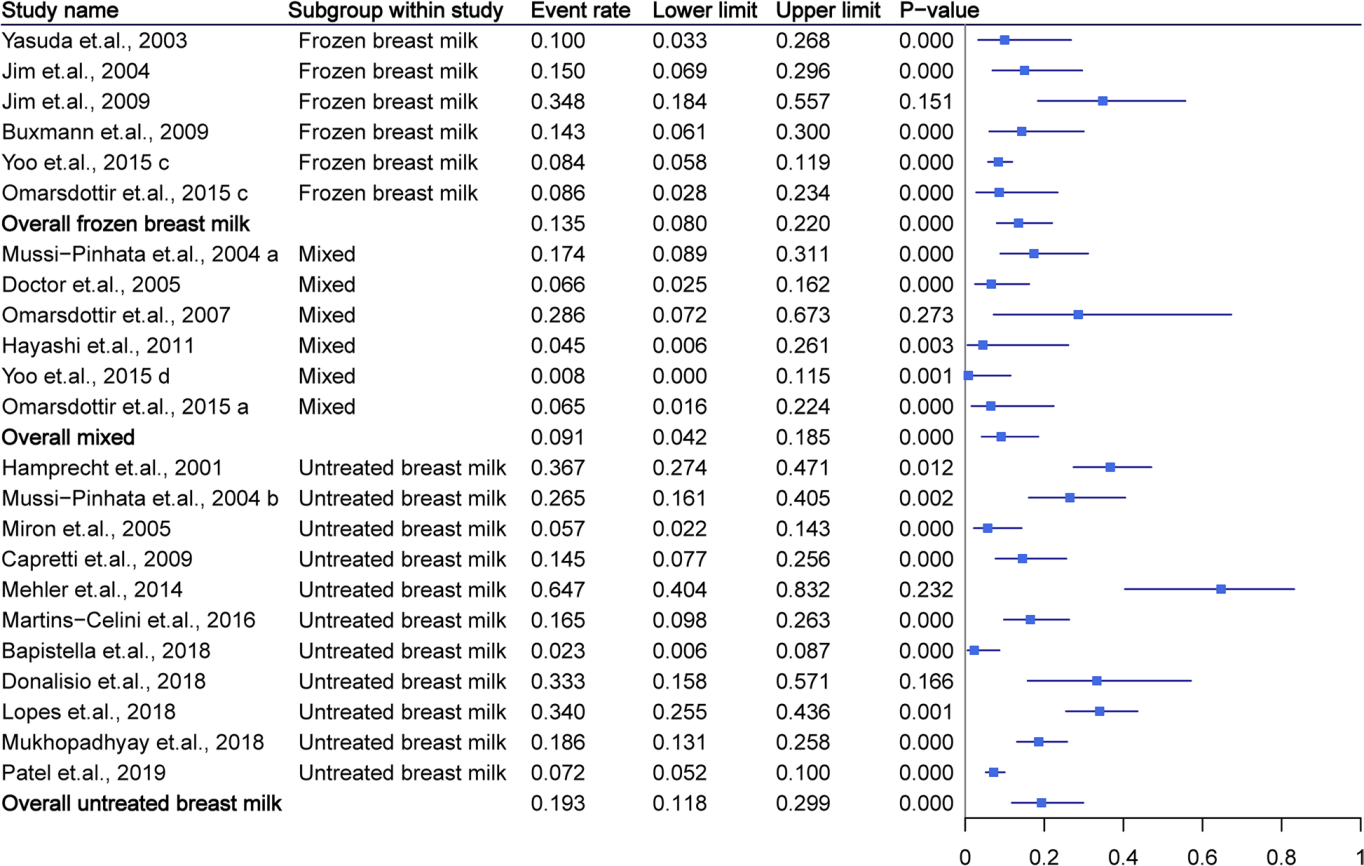
Summary of cytomegalovirus infection rate as grouped by breast milk type

Of the 12 studies that reported infants fed untreated breast milk, 1205 infants were included. The rate of CMV infection in each study ranged from 0 to 64%. The pooled rate of CMV infection for infants fed untreated breast milk was 19.3% (95% CI = 11.8–29.9%, *P* < 0.01), and showed no significant heterogeneity (*I*^*2*^ = 37%). For infants fed frozen breast milk, 52 CMV-positive infants were included. The pooled CMV infection rate (13.5, 95% CI = 8.0–22.0%, *P* < 0.01, *I*^*2*^ = 46%) was significantly lower than that of infants fed untreated breast milk (overall *P* < 0.01). Seven studies with 768 infants reported the result on infants fed mixed milk. The pooled CMV infection rate (9.1, 95% CI = 4.2–18.5%, *P* < 0.01, *I*^*2*^ = 30%) was similar to the infants who were fed frozen breast milk and were significantly lower than that of infants fed untreated breast milk (*P* < 0.01). The pooled CMV infection rate for all infants was 14.4% (95% CI = 10.1–20.2%, *P* < 0.01, *I*^*2*^ = 40%). The detailed data and funnel plot are shown in Fig. 2 and Supplementary Fig. 2.

### CMV-related symptoms

**Fig. 3:**
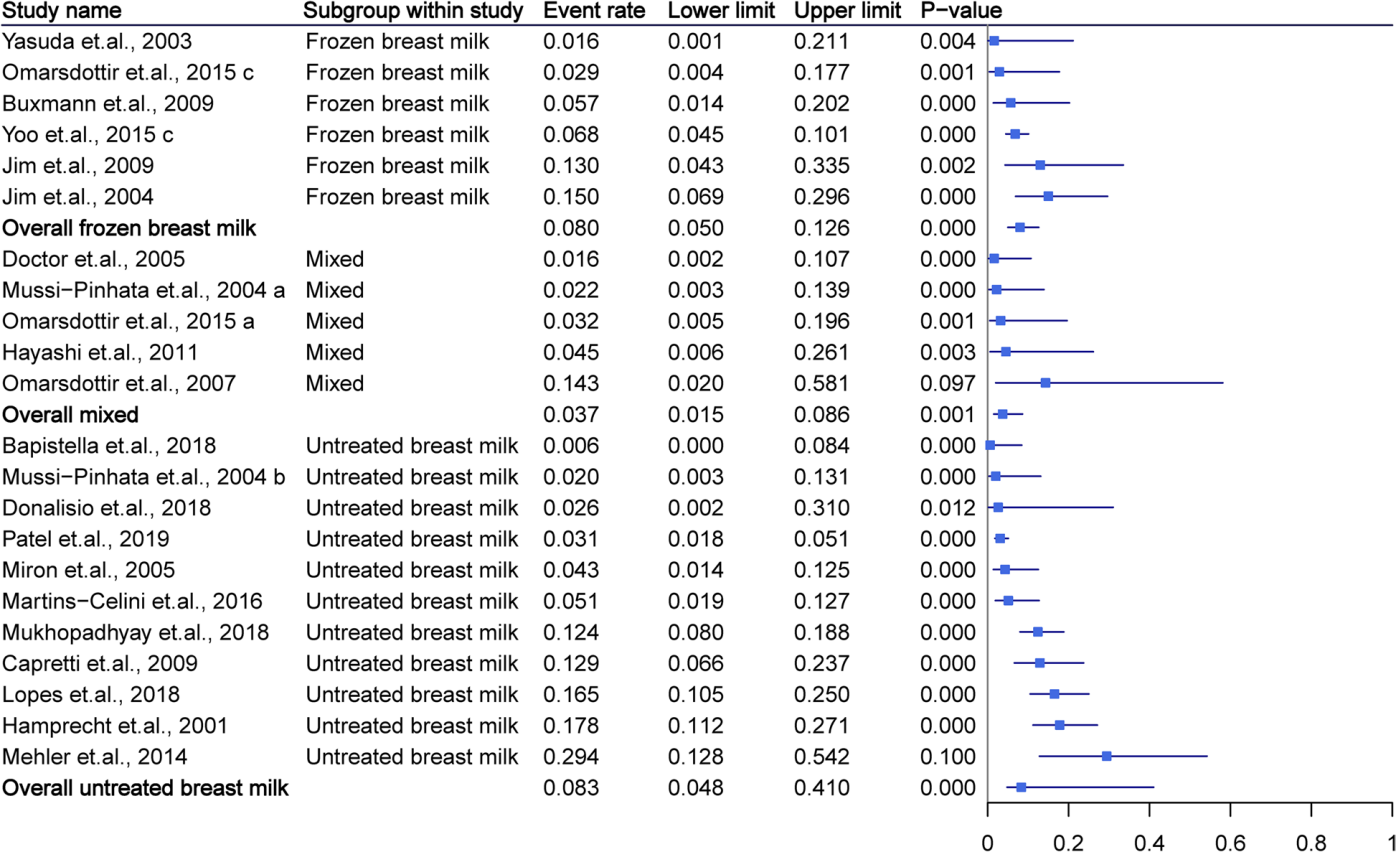
Summary of cytomegalovirus-related symptoms as grouped by breast milk type. The mixed group had a significantly lower rate of symptoms than other groups (*P* < 0.01)

The included studies mainly reported CMV-related symptoms such as thrombocytopenia, neutropenia, hepatitis, hepatosplenomegaly, and elevated liver enzymes, sequentially. The pooled symptom rates for infants fed untreated breast milk, frozen breast milk, and mixed feeding were 8.3% (95% CI = 4.8–14.0%, *P* < 0.01), 8.0% (95% CI = 5.0–12.6%, *P* < 0.01), and 3.7% (95% CI = 1.5–8.6%, *P* < 0.01), respectively. The pooled overall rate was 7.5% (95% CI = 5.1–10.7%, *P* < 0.01). The mixed group had a significantly lower rate of symptoms than the other groups (*P* < 0.01). All pooled results demonstrated no significant heterogeneity (all *I*^*2*^ < 50%). More detailed data were summarized in Fig. 3.

### CMV-SLS

**Fig. 4:**
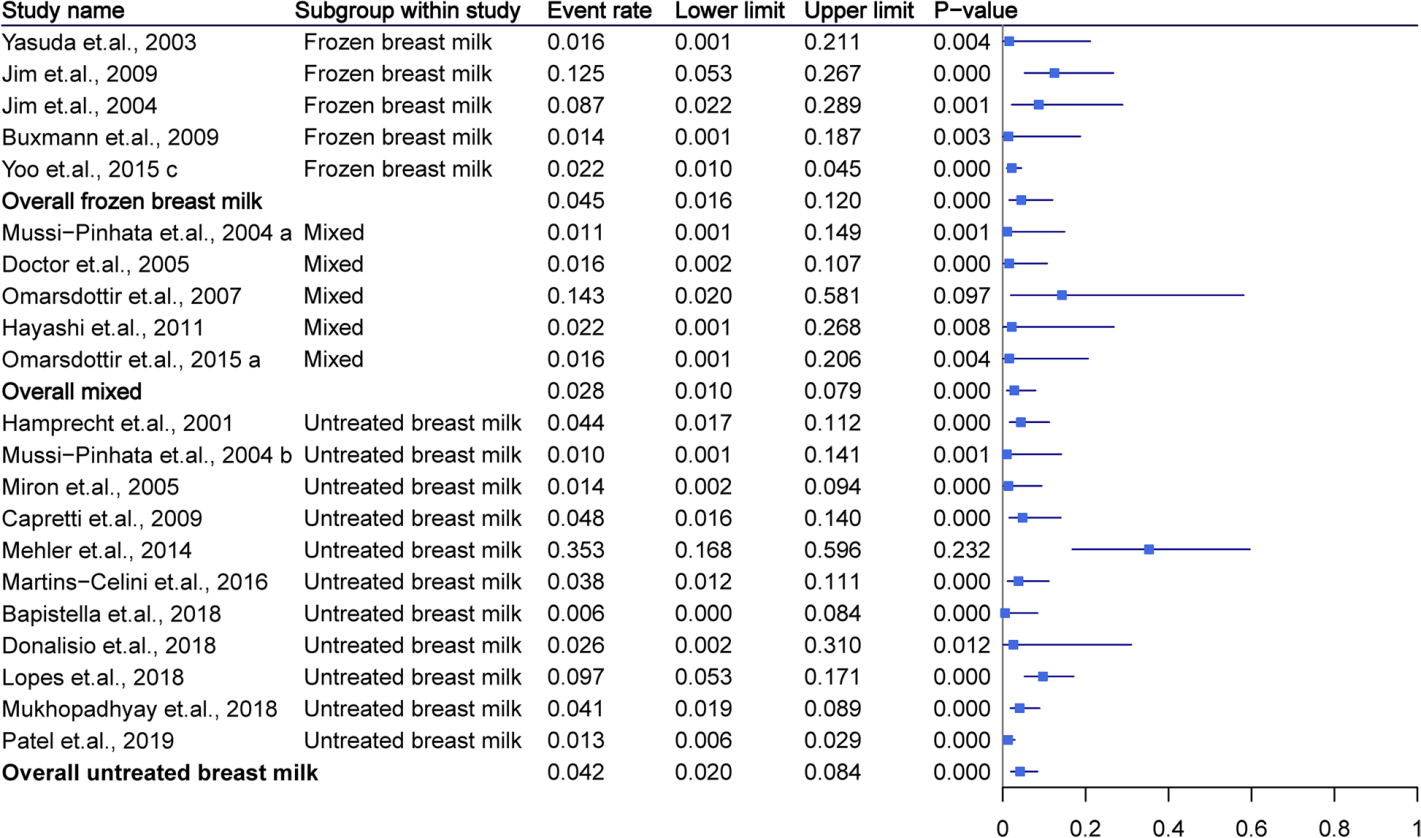
Summary of cytomegalovirus sepsis-like syndrome as grouped by breast milk type. The mixed feeding group demonstrated a significantly lower rate than the other two groups (*P* < 0.01)

Twenty-two studies with 2362 patients provided data on CMV-SLS. The pooled rate or infants fed untreated breast milk was 4.2% (95% CI = 2.0–8.4%, *P* < 0.01) and showed lower heterogeneity (*I*^2^ = 30%). The detailed data are presented in Fig. 4. The mixed feeding group demonstrated a significantly lower rate than the other two groups (*P* < 0.01).

### Meta-regression analyses

Meta-regression analyses were performed for the types of milk, NOS score, means of gestational ages, and study design. The types of milk were significantly associated in a gradient fashion (*P* < 0.0001, Supplementary Fig. 3). However, no significant gradient associations were observed for the other outcomes.

### Publication bias

No potential publication bias among the included trials was observed according to Begg’s rank correlation analysis and Egger’s weighted regression analysis (all *P* > 0.05, Supplementary Table 4).

## Discussion

In the current meta-analysis, 21 studies with a total of 1920 LBW and premature infants were included. All included studies were of relatively high quality. The pooled rate for infants fed untreated and frozen breast milk was 19.3 and 13.5%, respectively. LBW or preterm infants in the untreated breast milk group were associated with a significantly higher CMV infection rate via breast milk when compared to other feeding groups.

Comparing with the previous meta-analysis [11], we included more studies (11 vs. 21) with more infants. However, similarly, the infants infected with CMV via breast milk were rare. CMV infection via breast milk does not usually occur in full-term infants due to the transmission of protective maternal antibodies. A previous study reported the transmission process of maternal antibodies, which start within 29 gestational weeks [35]. For LBW or premature infants, this transmission of antibodies from the mothers might be absent, putting the infants at high risk for CMV infection postnatally [35, 36]. Therefore, premature infants, especially LBW infants, are associated with symptomatic and even severe CMV infection [37]. It has been reported that milk whey contains viable CMV virions due to different breast milk compartments. For the mothers who were CMV positive for milk, many of them were seropositive. A previous study has demonstrated the predominant role of cell-free virus transmission by breast milk [38]. While the risk of postnatal CMV infection is inversely proportional to GA, several studies have reported a strong association between high CMV viral load in bone marrow cells and risk of transmission [12, 29], as well as an inverse correlation between CMV-specific IgG avidity in breast milk and CMV load [32].

Compared to term infants, preterm infants, especially high-risk preterm infants, have more immature organ development and are more likely to suffer from series of morbidities such as pulmonary, hematologic, or hepatic conditions. Thus, CMV infections likely cause damage to the developing organs, and its transmission via breast milk might be a cofactor in aggravating the clinical course of pre-existing diseases and neurological implications in certain preterm infants. CMV-SLS has been introduced to describe the severe symptoms associated with CMV infection in LBW or premature infants. In the current study, CMV transmission via breast milk that led to severe symptomatic infections (such as SLS) in infected infants was rare. The data on long-term sequelae, such as neurological and cognitive sequelae, and sensorineural hearing loss, was insufficient.

Breast milk is considered an optimal food for preterm infants due to its benefits in preventing many comorbidities such as necrotizing enterocolitis (NEC) and retinopathy of prematurity (ROP) and improving neurodevelopmental outcomes. NEC is an acute inflammatory reaction and is the leading gastrointestinal cause of morbidity and mortality in preterm infants [39]. Studies have shown that raw breast milk nutrition significantly reduces the risk of NEC [40]. However, the link between NEC and postnatal CMV is controversial [40]. Fresh breast milk contains beneficial factors, but the risk of CMV transmission to cause severe systematic symptoms cannot be neglected. In this meta-analysis, frozen breast milk or mixed feeding protocol were associated with a lower risk of CMV-related infection and injury. The prospective study conducted by Capretti et al. [28] reported that CMV infection though the mother’s milk neither influenced short-term outcome or length of hospital stay nor did it cause long-term neurosensorial sequelae. Therefore, the benefits of fresh milk might overweight the risks related to CMV transmission for most LBW infants. However, currently, in the clinical settings, the breast milk feeding practices vary in different centers as many experts favor frozen breast milk for feeding in high-risk preterm infants. This perspective seems to be consistent with our study, which indicates frozen breast milk or mixed feeding are in priority for LBW or preterm infants. As comprehensive long-term follow up data on the impact of postnatal CMV infection have not yet established. The data from this kind of studies remain critical for future analysis and assists in constructing an optimal breast milk feeding practice in different countries based on eligible infants.

Although most included studies were of high quality (NOS ≥6 scores), it is necessary to consider the limitations of the present meta-analysis when interpreting the results. Firstly, most of the studies included few participants. Due to the limited number of patients included in each study, it was difficult to perform subgroup or sensitivity analyses. Moreover, the representativeness of the target population might be weakened. Secondly, the inclusion criteria for each study varied. For example, most studies included infants with birth weight lower than 1200 g or GA earlier than 32 weeks, while others included infants lower than 1500 g or GA earlier than 35 weeks. Moreover, some studies only considered birth weight as the inclusion criteria. Thus, the definition for the targeted population might cause heterogeneity and reduce the stability of the results. Thirdly, the number of the included studies was limited, and most of them were conducted in western countries and focused on Caucasians. So, the results might be affected by the living factors, environmental factors, medical level, and genetic factors. Therefore, the results in the current setting can only partly annotate the associations. Fourthly, a relative effect measure such as the odds ratio would be more informative. However, due to the insufficient information provided by the included studies, we cannot implement that. Fifthly, potential language bias might exist because our literature searches considered only articles published in English. Lastly, more details on the duration of breastfeeding and the proportion of raw breast milk in the mixed feeding group require clarification. Their impact on the transmission of CMV via breast milk should be stratified in further investigation.

## Conclusions

This meta-analysis provided pooled results based on 21 studies from 11 different regions or countries and summarized a data set of 1920 infants. The infants fed with untreated breast milk had a significantly higher CMV infection rate via breast milk in LBW or premature infants compared to other groups. The mixed feeding group had a significantly lower rate of CMV-related symptoms and CMV-SLS than the other groups. In the future, more original studies conducted in different counties and ethnic cultures are warranted to verify the results reported in this study.

## Supplementary Information

**Additional file 1 **: **Supplementary Figure 1.** Summary of cytomegalovirus infection rate in all included mothers.

**Additional file 2 **: **Supplementary Figure 2.** Funnel plot for the summary of overall cytomegalovirus infection rate.

**Additional file 3 **: **Figure S3.**

**Additional file 4 **: **Supplementary Table 1**. Literature search strategy.

**Additional file 5 **: **Supplementary Table 2**. Characteristics of study population.

**Additional file 6 **: **Supplementary Table 2**. Publication bias of summarized outcomes. **Supplementary Table 3**. Quality assessment of included studies by Newcastle-Ottawa Scale.

**Additional file 7 **: **Supplementary Table 4**. Publication bias of summarized outcomes.

## Data Availability

The datasets used and/or analyzed during the current study are available from the corresponding author on reasonable request.

## Abbreviations

CMV: Cytomegalovirus
LBW: Low birth weight
CMV-SLS: CMV-related sepsis-like syndrome
CI: Confidence interval
PCR: Polymerase chain reaction
NOS: Newcastle-Ottawa Scale
CIs: Confidence intervals
GA: Gestational age
BW: Body weight
NEC: Necrotizing enterocolitis
ROP: Retinopathy of prematurity
