# Supplementary figures and images for: Transmission of cytomegalovirus via breast milk in low birth weight and premature infants: a systematic review and meta-analysis
Source: BMC Pediatr. 2021 Nov 22;21:520. doi: 10.1186/s12887-021-02984-7 (PMC8607598; doi:10.1186/s12887-021-02984-7)

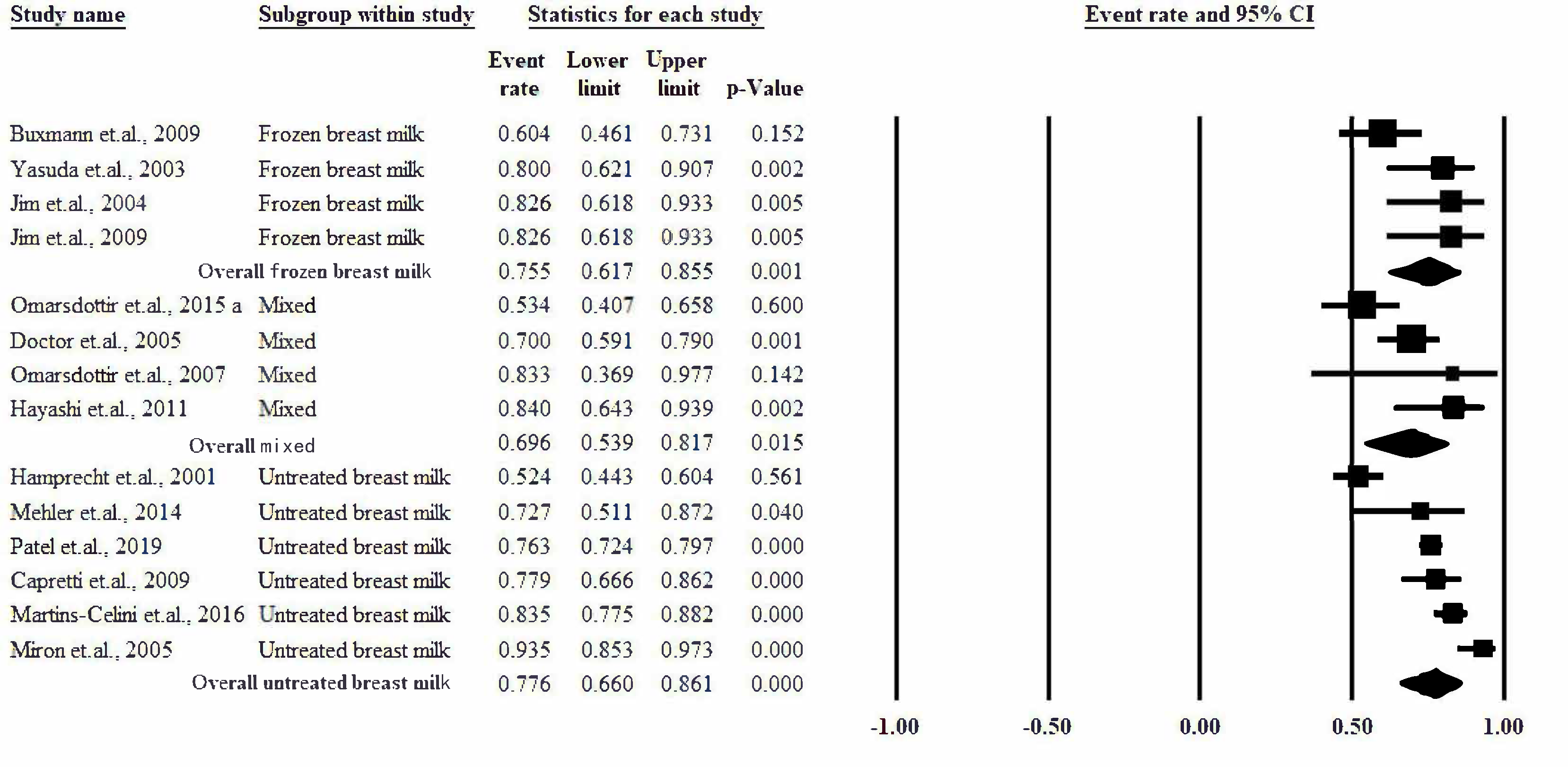

Supplement: Supplementary file 1 — Additional file 1 : Supplementary Figure 1. Summary of cytomegalovirus infection rate in all included mothers. [file 12887_2021_2984_MOESM1_ESM.tif]

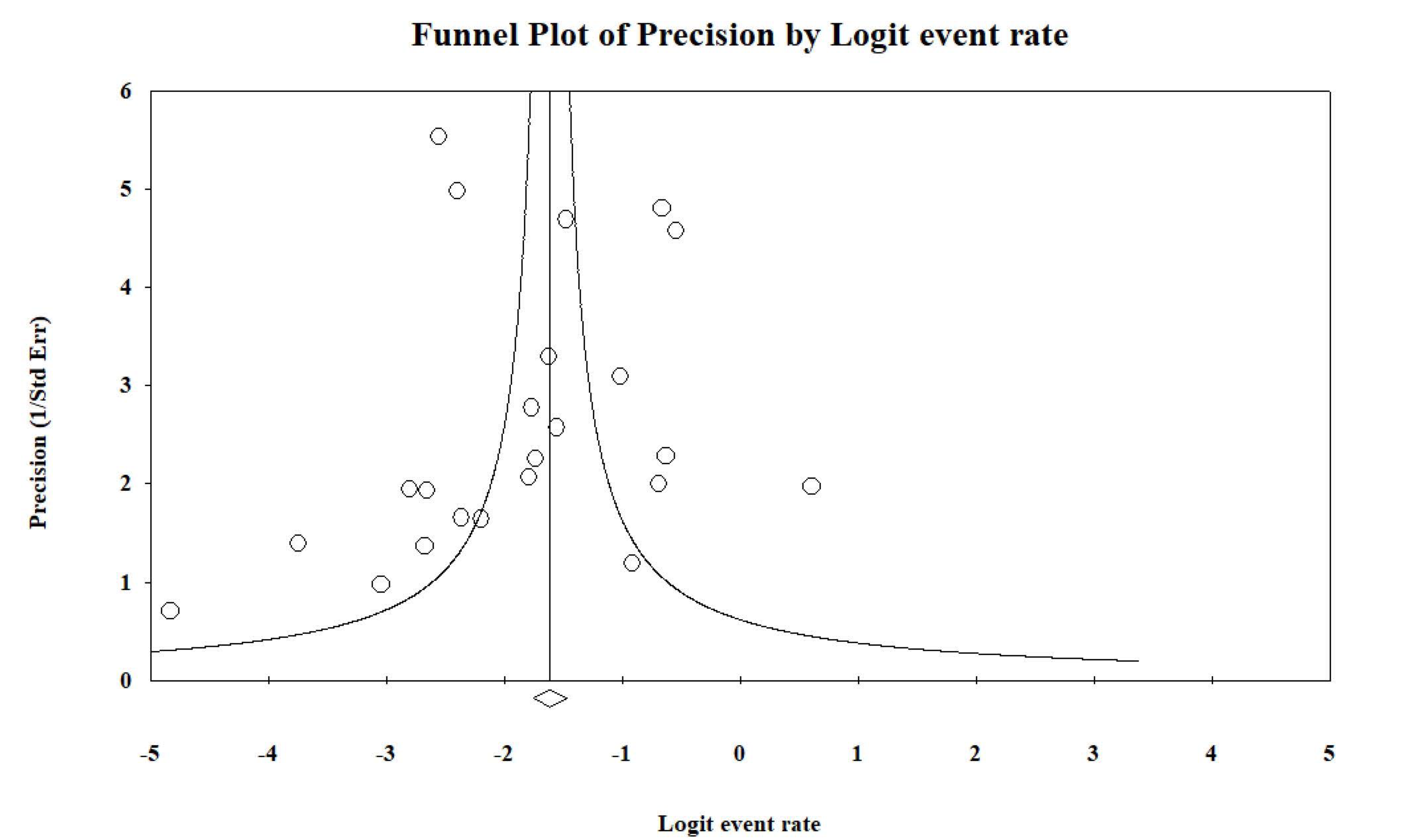

Supplement: Supplementary file 2 — Additional file 2 : Supplementary Figure 2. Funnel plot for the summary of overall cytomegalovirus infection rate. [file 12887_2021_2984_MOESM2_ESM.tif]

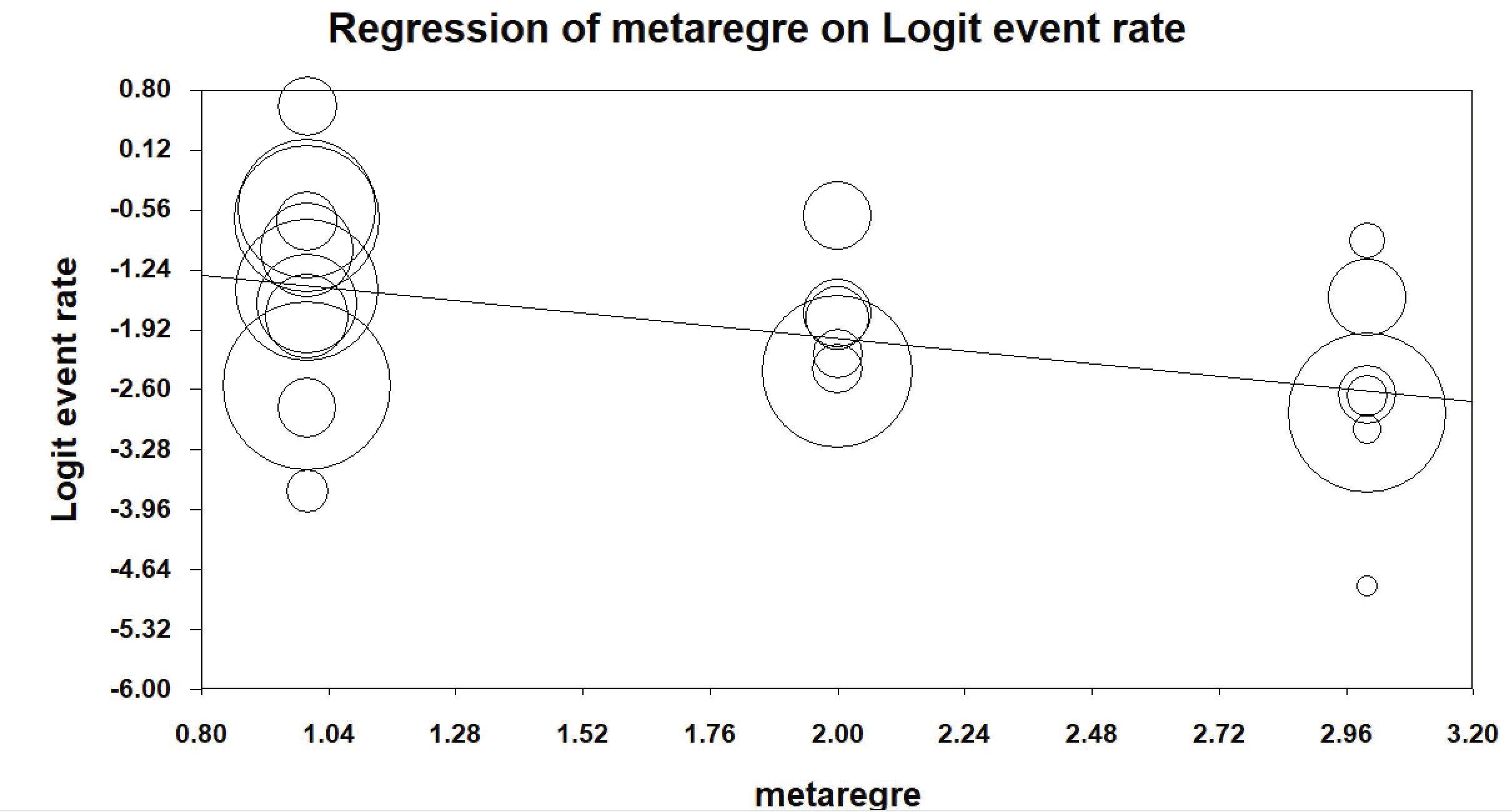

Supplement: Supplementary file 3 — Additional file 3 : Figure S3. [file 12887_2021_2984_MOESM3_ESM.tif]
