# Supplementary material for: Transmission of cytomegalovirus via breast milk in low birth weight and premature infants: a systematic review and meta-analysis
Source: BMC Pediatr. 2021 Nov 22;21:520. doi: 10.1186/s12887-021-02984-7 (PMC8607598; doi:10.1186/s12887-021-02984-7)
Supplement: Supplementary file 4 — Additional file 4 : Supplementary Table 1. Literature search strategy. [file 12887_2021_2984_MOESM4_ESM.docx]

**Supplementary Table 1. Literature search strategy**

| 1#: "Breast Feeding"[Mesh] OR "Breast Feeding" |
| --- |
| 2#: "Milk, Human"[Mesh] OR “breast milk” |
| 3#: "Premature Birth"[Mesh] OR “preterm” |
| 4#: "Infant, Low Birth Weight"[Mesh] OR “low birth weight infants” OR “low birth weight infant” OR “LBW” OR “preterm infants” |
| 5#: "Cytomegalovirus"[Mesh] OR “Cytomegalovirus” OR “CMV” |
| 6#: 1 OR 2 |
| 7#: 3 OR 4 |
| 8#: 6 AND 7 AND 5 |
