# Supplementary material for: Transmission of cytomegalovirus via breast milk in low birth weight and premature infants: a systematic review and meta-analysis
Source: BMC Pediatr. 2021 Nov 22;21:520. doi: 10.1186/s12887-021-02984-7 (PMC8607598; doi:10.1186/s12887-021-02984-7)
Supplement: Supplementary file 5 — Additional file 5 : Supplementary Table 2. Characteristics of study population. [file 12887_2021_2984_MOESM5_ESM.docx]

**Supplementary Table 2. Characteristics of study population**

| **Study included** | **Country** | **Study design** | **Infants fed** | **Inclusion Criteria**  **(BW, weeks/GA, grams)** | **CMV Methods** | | **Infant weight (gram, means±SD)** |
| --- | --- | --- | --- | --- | --- | --- | --- |
|  |  |  |  |  | **Mothers** | **Infants** |  |
| Hamprecht et.al., 2001 | Germany | prospective | Untreated breast milk | <1500 or <32 | PCR, culture, serology | PCR, culture, serology | NA |
| Yasuda et.al., 2003 | Japan | prospective | Frozen breast milk | <1200 or <34 | rt-PCR | rt-PCR | NA |
| Jim et.al., 2004 | Taiwan | prospective | Frozen breast milk | <1500 and <35 | PCR | PCR, culture, serology | 1300±200 |
| Mussi-Pinhata et.al., 2004 | Brazil | prospective | Untreated breast milk | <1500 or <34 | PCR | PCR | 1170 (710-2165)^c^ |
| Doctor et.al., 2005 | Canada | prospective | Mixed | <1000 or <28 | serology | immunofluorescence | 771±134 |
| Meier et.al., 2005 | Germany | prospective | Untreated breast milk | ≤2100 or <33 | PCR, culture, serology | PCR, culture, serology | 1119 (380-2010) ^c^ |
| Miron et.al., 2005 | Israel | prospective | Untreated breast milk | <1500 or <32 | PCR, culture, serology | PCR, culture, serology | 1242±230 |
| Omarsdottir et.al., 2007 | Sweden | prospective | Mixed | ≤1166 or <28 | serology | PCR, culture, serology | 892 (604-1166) ^c^ |
| Capretti et.al., 2009 | Italy | prospective | Untreated breast milk | <1500 and <32 | PCR, culture, serology | PCR, culture, serology | 1125±277 |
| Jim et.al., 2009 | Taiwan | prospective | Frozen breast milk | <1500 and <35 | RT-PCT | RT-PCT | NA |
| Buxmann et.al., 2009 | Germany | prospective | Frozen breast milk | <1710 or <31 | RT-PCT | RT-PCT | 1030 (600-1710) ^c^ |
| Hayashi et.al., 2011 | Japan | prospective | Mixed | <1000 or <28 | rt-PCR | rt-PCR | 802 (512-1108) ^c^ |
| Mehler et.al., 2014 | Germany | prospective | Untreated breast milk | <28 ^a^ | PCR, culture, serology | PCR, culture, serology | 541 (390-713) ^c^ |
|  |  |  |  |  |  |  |  |
| Yoo et.al., 2015 | Korea | retrospective | Mixed | <1000 ^b^ | NA | Serology, PCR | 711±162 |
| Omarsdottir et.al., 2015 | Sweden | prospective | Mixed | <28 ^a^ | rt-PCR | rt-PCR | 846±178 |
| Martins-Celini et.al., 2016 | Brazil | prospective | Untreated breast milk | <30 ^a^ | Serology, PCR | Serology, PCR | 748±196 |
| Mukhopadhyay et.al., 2018 | United states | retrospective | Untreated breast milk | <1500 ^b^ | Serology, PCR | Serology, PCR | 822±260 |
| Patel et.al., 2019 | United states | prospective | Untreated breast milk | <1500 ^b^ | Serology, PCR | Serology, PCR | 981±254 |

Abbreviations: CMV, cytomegalovirus; PCR, polymerase chain reaction; rt-PCR, real-time polymerase chain reaction; GA, gestational age; BW, body weight; SD, standard deviation; NA, not available.

^a^, gestational age.

^b^, body weight.

^c^, median and range.
