# Supplementary material for: Transmission of cytomegalovirus via breast milk in low birth weight and premature infants: a systematic review and meta-analysis
Source: BMC Pediatr. 2021 Nov 22;21:520. doi: 10.1186/s12887-021-02984-7 (PMC8607598; doi:10.1186/s12887-021-02984-7)
Supplement: Supplementary file 6 — Additional file 6 : Supplementary Table 2. Publication bias of summarized outcomes. Supplementary Table 3. Quality assessment of included studies by Newcastle-Ottawa Scale. [file 12887_2021_2984_MOESM6_ESM.docx]

**Supplementary Table 3. Quality assessment of included studies by Newcastle-Ottawa Scale**

| **Study included** | **Representativeness**  **of the**  **exposed**  **cohort** | **Selection**  **of the exposed cohort** | **Selection**  **of the non**  **exposed**  **cohort** | **Demonstration that the outcome of interest was not present at the start of the study** | **Comparability of**  **cohorts on**  **the basis of**  **the design**  **or analysis** | **Assessment of**  **outcome** | **Was followed-up long enough for**  **outcomes**  **to occur** | **Adequacy of follow-up of cohorts** | **Overall quality score** |
| --- | --- | --- | --- | --- | --- | --- | --- | --- | --- |
| Hamprecht et.al., 2001 | - | ☆ | ☆ | ☆☆ | ☆ | ☆ | ☆ | ☆ | 8 |
| Yasuda et.al., 2003 | ☆ | ☆ | ☆ | ☆ | ☆ | ☆ | ☆ | - | 7 |
| Jim et.al., 2004 | - | ☆ | ☆ | ☆ | ☆ | ☆ | ☆ | - | 6 |
| Mussi-Pinhata et.al., 2004 | - | ☆ | ☆ | ☆ | ☆ | ☆ | ☆ | ☆ | 7 |
| Doctor et.al., 2005 | - | ☆ | ☆ | ☆ | ☆ | ☆ | ☆ | ☆ | 7 |
| Meier et.al., 2005 | - | ☆ | ☆ | ☆☆ | ☆ | ☆· | ☆ | - | 6 |
| Miron et.al., 2005 | ☆ | ☆ | ☆ | ☆ | ☆ | ☆ | ☆ | ☆ | 8 |
| Omarsdottir et.al., 2007 | ☆ | ☆ | ☆ | ☆ | ☆ | ☆ | ☆ | - | 7 |
| Capretti et.al., 2009 | - | ☆ | ☆ | ☆ | ☆ | ☆ | ☆ | ☆ | 7 |
| Jim et.al., 2009 | - | ☆ | ☆ | ☆☆ | ☆ | ☆ | ☆ | - | 7 |
| Buxmann et.al., 2009 | - | ☆ | ☆ | ☆☆ | ☆ | ☆ | ☆ | ☆ | 8 |
| Hayashi et.al., 2011 | - | ☆ | ☆ | ☆ | ☆ | ☆ | ☆ | ☆ | 7 |
| Mehler et.al., 2014 | ☆ | ☆ | ☆ | ☆☆ | ☆ | ☆ | ☆ | - | 8 |
|  |  |  |  |  |  |  |  |  |  |
| Yoo et.al., 2015 | ☆ | ☆ | ☆ | ☆ | ☆ | ☆ | ☆ | - | 7 |
| Omarsdottir et.al., 2015 | - | ☆ | ☆ | ☆☆ | ☆ | ☆ | ☆ | ☆ | 8 |
| Martins-Celini et.al., 2016 | ☆ | ☆ | ☆ | ☆☆ | ☆ | ☆ | ☆ | - | 8 |
| Mukhopadhyay et.al., 2018 | - | ☆ | ☆ | ☆ | ☆ | ☆ | ☆ | ☆ | 7 |
| Patel et.al., 2019 | - | ☆ | ☆ | ☆☆ | ☆ | ☆ | ☆ | - | 7 |

**Supplementary Table 2. Publication bias of summarized outcomes**

| **Outcomes** | **Begg (*P* value)** | **Egger (*P* value)** |
| --- | --- | --- |
| Summarized cytomegalovirus rate for all included mothers | 0.23 | 0.99 |
| Summarized cytomegalovirus rate for untreated breast milk group | 0.76 | 0.62 |
| Summarized cytomegalovirus rate for frozen breast milk group | 0.41 | 0.39 |
| Summarized cytomegalovirus rate for mixed milk group | 0.45 | 0.28 |
| Summarized cytomegalovirus rate for all infants | 0.68 | 0.42 |
| Summarized cytomegalovirus symptoms for untreated breast milk group | 0.23 | 0.11 |
| Summarized cytomegalovirus symptoms for frozen breast milk group | 0.89 | 0.21 |
| Summarized cytomegalovirus symptoms for mixed milk group | 0.23 | 0.65 |
| Summarized cytomegalovirus symptoms for all infants | 0.35 | 0.17 |
| Summarized cytomegalovirus sepsis-like syndrome for untreated breast milk group | 0.86 | 0.16 |
| Summarized cytomegalovirus sepsis-like syndrome for frozen breast milk group | 0.09 | 0.15 |
| Summarized cytomegalovirus sepsis-like syndrome for mixed milk group | 0.53 | 0.12 |
| Summarized cytomegalovirus sepsis-like syndrome for all infants | 0.75 | 0.66 |
