# Supplementary material for: Transmission of cytomegalovirus via breast milk in low birth weight and premature infants: a systematic review and meta-analysis
Source: BMC Pediatr. 2021 Nov 22;21:520. doi: 10.1186/s12887-021-02984-7 (PMC8607598; doi:10.1186/s12887-021-02984-7)
Supplement: Supplementary file 7 — Additional file 7 : Supplementary Table 4. Publication bias of summarized outcomes. [file 12887_2021_2984_MOESM7_ESM.docx]

**Supplementary Table 4. Publication bias of summarized outcomes**

| **Outcomes** | **Begg (*P* value)** | **Egger (*P* value)** |
| --- | --- | --- |
| Summarized cytomegalovirus rate for all included mothers | 0.20 | 0.79 |
| Summarized cytomegalovirus rate for untreated breast milk group | 0.70 | 0.60 |
| Summarized cytomegalovirus rate for frozen breast milk group | 0.41 | 0.39 |
| Summarized cytomegalovirus rate for mixed milk group | 0.40 | 0.69 |
| Summarized cytomegalovirus rate for all infants | 0.68 | 0.42 |
| Summarized cytomegalovirus symptoms for untreated breast milk group | 0.13 | 0.09 |
| Summarized cytomegalovirus symptoms for frozen breast milk group | 0.89 | 0.21 |
| Summarized cytomegalovirus symptoms for mixed milk group | 0.49 | 0.20 |
| Summarized cytomegalovirus symptoms for all infants | 0.35 | 0.17 |
| Summarized cytomegalovirus sepsis-like syndrome for untreated breast milk group | 0.86 | 0.16 |
| Summarized cytomegalovirus sepsis-like syndrome for frozen breast milk group | 0.09 | 0.15 |
| Summarized cytomegalovirus sepsis-like syndrome for mixed milk group | 0.29 | 0.21 |
| Summarized cytomegalovirus sepsis-like syndrome for all infants | 0.75 | 0.66 |
